# The N-Terminal Sequence of Prion Protein Consists an Epitope Specific to the Abnormal Isoform of Prion Protein (PrP^Sc^)

**DOI:** 10.1371/journal.pone.0058013

**Published:** 2013-02-28

**Authors:** Kentaro Masujin, Yuko Kaku-Ushiki, Ritsuko Miwa, Hiroyuki Okada, Yoshihisa Shimizu, Kazuo Kasai, Yuichi Matsuura, Takashi Yokoyama

**Affiliations:** 1 Prion Disease Research Center, National Institute of Animal Health, Tsukuba, Ibaraki, Japan; 2 Nippi Research Institute of Biomatrix, Toride, Ibaraki, Japan; Ohio State University, United States of America

## Abstract

The conformation of abnormal prion protein (PrP^Sc^) differs from that of cellular prion protein (PrP^C^), but the precise characteristics of PrP^Sc^ remain to be elucidated. To clarify the properties of native PrP^Sc^, we attempted to generate novel PrP^Sc^-specific monoclonal antibodies (mAbs) by immunizing PrP-deficient mice with intact PrP^Sc^ purified from bovine spongiform encephalopathy (BSE)-affected mice. The generated mAbs 6A12 and 8D5 selectivity precipitated PrP^Sc^ from the brains of prion-affected mice, sheep, and cattle, but did not precipitate PrP^C^ from the brains of healthy animals. In histopathological analysis, mAbs 6A12 and 8D5 strongly reacted with prion-affected mouse brains but not with unaffected mouse brains without antigen retrieval. Epitope analysis revealed that mAbs 8D5 and 6A12 recognized the PrP subregions between amino acids 31–39 and 41–47, respectively. This indicates that a PrP^Sc^-specific epitope exists in the N-terminal region of PrP^Sc^, and mAbs 6A12 and 8D5 are powerful tools with which to detect native and intact PrP^Sc^. We found that the ratio of proteinase K (PK)-sensitive PrP^Sc^ to PK-resistant PrP^Sc^ was constant throughout the disease time course.

## Introduction

Transmissible spongiform encephalopathies (TSEs), or prion diseases, are fatal neurodegenerative disorders. They include scrapie in sheep and goat, bovine spongiform encephalopathy (BSE) in cattle, and Creutzfeldt-Jakob disease in humans [1]. TSEs are characterized by the accumulation of an abnormal isoform of prion protein (PrP^Sc^), which is generated by post-translational modification of the host-encoded cellular prion protein (PrP^C^). According to the protein-only hypothesis, PrP^Sc^ is the principal component of the infectious agent, the prion.

Conversion from PrP^C^ to PrP^Sc^ is a key event in the pathogenesis of prion diseases. PrP^Sc^ has a larger number of β-sheets and a lower α-helical structure content than PrP^C^ [2]–[4]. These conformational changes facilitate the aggregation of PrP^Sc^ and underlie its biochemical characteristics, such as insolubility to detergents and resistance to protease digestion [5], [6]. PrP^Sc^’s insolubility and tendency to aggregate have prevented its structural analysis. Although several PrP^Sc^ models have been proposed [7]–[9], the conformation of native PrP^Sc^ is still obscure.

To discriminate PrP^Sc^ from PrP^C^, proteinase K (PK) digestion has been widely used. This process completely digests PrP^C^, whereas the C-terminal region of truncated PrP^Sc^ remains, and these fragments are referred to as PrPcore, which is the only known disease-specific marker [10]. However, occasionally prion infectivity is not associated with a detectable PrPcore [11]. In addition, PrP^Sc^ has been reported to consist not only of a protease-resistant isoform but also of a protease-sensitive isoform [12]–[14]. These observations suggest an association between the PK-sensitive PrP^Sc^ (PrP^Sc^-sen) and prion infectivity [11]–[14]. Characterization of PrP^Sc^-sen is important for understanding prion pathogenesis. However, PK digestion destroys not only the partial PrP^Sc^ structure, but also the PrP^Sc^-sen molecule, and the precise properties of native PrP^Sc^, including PrP^Sc^-sen, remain to be elucidated. Analysis of native and intact PrP^Sc^ is necessary to clarify the role of PrP^Sc^-sen in prion pathogenesis.

PrP^Sc^-specific monoclonal antibodies (mAbs) are powerful tools for the analysis and isolation of the native PrP^Sc^. Several PrP^Sc^-specific monoclonal antibodies (mAbs) have been reported [15]–[21]. These mAbs distinguish PrP^Sc^ from PrP^C^ without PK digestion. By using these mAbs, several PrP^Sc^-specific epitopes have been identified. Interestingly, these mAbs recognize different sub-regions or sequences as their epitopes. A panel of PrP^Sc^-specific mAbs will help elucidate the properties of native PrP^Sc^.

Here, we generated novel PrP^Sc^-specific mAbs to analyze the properties of native and intact PrP^Sc^. PrP-deficient mice (PrP^0/0^) were immunized with intact PrP^Sc^ purified from BSE prion-affected mice. We examined the dynamics of native PrP^Sc^ in prion-affected mice during the course of the disease using the generated antibodies.

## Materials and Methods

### Ethics Statement

Procedures involving animal subjects have been approved by the Institute Animal Care and Use Committee at the National Institute of Animal Health (approval ID: 09–44, 10-005 and 11-008).

### Brain Samples

Mouse-passaged BSE and mouse-passaged scrapie strains ME7 and Chandler were used. These prions were routinely maintained by serial passage into wild-type mice (ICR; Japan SLC, Inc.) as described previously [22]–[24]. Brain samples from classical BSE (C-BSE) and scrapie-affected sheep were also used [25], [26]. Brains from diseased animals were removed and stored at −80°C for biochemical analysis or frozen in liquid nitrogen for histopathology.

### Purification of Intact PrP^Sc^

Intact PrP^Sc^ was purified from the brains of BSE-affected mice in accordance with a protocol reported previously with minor modifications [20]. In brief, the brains of BSE-affected mice were homogenized in buffer containing 10% (w/v) sarkosyl, 10 mM Tris-HCl (pH 8.3), 1 mM EDTA, 133 mM NaCl, 1 mM DTT, 0.4 M 4-(2-aminoethyl) benzenesulfonyl fluoride hydrochloride (Pefabloc; Roche Diagnostics), and a protease inhibitor cocktail (Roche Diagnostics) using a multi-bead shocker (Yasui Kikai) and centrifuged at 1,000×*g* for 10 min at 4°C. The supernatant was centrifuged at 180,000×*g* for 150 min at 4°C. The pellet was resuspended in buffer containing 10% NaCl, 1% Zwittergent 3–14 (Calbiochem), 10 mM Tris-HCl (pH 8.3), 1 mM EDTA, 133 mM NaCl, 1 mM DTT, and 0.4 M Pefabloc. The sample was centrifuged at 250,000×*g* for 90 min at 20°C after sonication. The pellet was resuspended in buffer containing 10 mM Tris-HCl (pH 7.4), 5 mM MgCl_2_, and 100 mM NaCl and subsequently incubated with RNase A (100 µg/mL) and DNase I (20 µg/mL) for 2 h at 37°C. The sample was mixed with 20 mM EDTA, 10% NaCl, and 1% Zwittergent 3–14, loaded onto a 1 M sucrose cushion with 100 mM NaCl and 0.5% sulfobetaine, and then centrifuged at 250,000×*g* for 90 min at 20°C. Finally, the pellet was resuspended in PBS. The purity of intact PrP^Sc^ was estimated using 12% sodium dodecyl sulfate (SDS)-polyacrylamide gel electrophoresis (PAGE) with silver staining. The concentration of intact PrP^Sc^ was estimated by BCA protein assay (Pierce).

### Production of Monoclonal Antibodies

Five-week-old PrP^0/0^ mice were immunized subcutaneously with 10 µg intact PrP^Sc^ in complete Freund’s adjuvant (Wako). The mice were boosted with 10 µg intact PrP^Sc^ in incomplete Freund’s adjuvant (Wako) twice at 14 day intervals. Three days before the cell fusion, mice were immunized intravenously with intact PrP^Sc^ in PBS. Splenocytes obtained from the immunized mice were fused with mouse myeloma cells (Sp2/0-Ag14, DS Pharma Biomedical) with the standard protocol [27]. The hybridoma culture supernatants were screened by enzyme-linked immunosorbent assay (ELISA). MAbs were purified using a MAb Trap Kit (GE Healthcare), and the mAb subclasses were determined using an IsoStrip Mouse Antibody Isotyping Kit (Roche Applied Science).

### Antibodies

The following mAbs against PrP were used in this study: mAb T2 [28], 6H4 (Prionics), 31C6 [29], and SAF32 (SPI-bio). MAb P2b (isotype: immunoglobulin (Ig) G2b), which recognizes a plant-derived protein, was used as negative control. MAb binding was detected by horseradish peroxidase (HPR)-conjugated anti-mouse IgG. In some experiments, HRP-conjugated mAb T2 was used.

### ELISA

The ELISA was performed according to a previously described method [20]. The ELISA plate (MaxiSorp, Nunc) was coated with Seprion ligand, which binds selectively to PrP^Sc^ (Microsens Biotechnologies) [30]. After plates were blocked with 5% fetal bovine serum (FBS) in PBS, 100 µL samples of 0.1% (w/v) brain homogenate from BSE-affected mice in buffer containing 5 mM Tris-HCl (pH 8.3), 10% (w/v) sarkosyl, and 10% (w/v) Triton X-100 were added to the plates and incubated for 2 h at room temperature (RT). Seprion ligand-coated ELISA plates were considered to predominantly bind PrP^Sc^. The plates were washed with Tris-buffered saline (TBS) containing 50 mM Tris-HCl and 0.3 M NaCl. The plates were then treated with or without 4 M guanidine thiocyanate (GdnSCN) to generate denatured PrP^Sc^ and native PrP^Sc^, respectively. The supernatant of each hybridoma was added to 2 wells (denatured and native PrP^Sc^) and incubated for 1 h at RT. After washing in TBS containing 0.05% Tween-20, plates were incubated with HRP-conjugated anti-mouse IgG. The immunocomplex was detected using 3, 3′, 5, 5′-tetramethylbenzidine (TMB) as the substrate and analyzed by a microplate reader at 450 nm wavelength. MAb 6H4 was used as a positive control for ELISA. The ELISA values were compared between native and denatured PrP^Sc^.

### Immunoprecipitation

Brain homogenate was mixed with buffer containing 0.01% Triton X-100, 0.01% sodium deoxycholate, 100 mM NaCl, 10 mM Tris-HCl (pH 7.6), protease inhibitor cocktail, and 0.4 M Pefabloc for a final concentration of 5% (w/v). After sonication, the sample was centrifuged at 500×*g* for 15 min at 4°C, and the supernatants were collected. The brain homogenates were diluted to final concentrations of 0.01–0.1% (w/v) with buffer containing 3% Tween 20 and 3% Triton X-100 in PBS. Protein-G Dynabeads (Dynal Biotech) were blocked with 4% (w/v) blocking solution (Block Ace; Yukijirushi). Brain homogenate samples (250 µL) were incubated with mAbs at concentrations of 1 µg/mL in 500 µL reaction volumes for 1 h at RT with rotation. Protein-G Dynabeads (20 µL) were then added and incubated for 1 h at RT on a rotator. The beads were washed 7 times with PBS containing 2% Tween 20 and 2% Triton X-100. To determine the PK resistance of the precipitated PrP, beads were treated with 50 µg/mL of Proteinase K (Roche Diagnostic) and incubated for 1 h at 37°C. PK digestion was terminated with 2 mM Pefabloc. The beads were resuspended in gel-loading buffer containing 2% (w/v) SDS and boiled for 10 min. Eluted PrP was detected by western blotting (WB). To examine the immunoreactivity of mAbs against PrPcore, brain homogenates were treated with 50 µg/mL of PK. Digestion was terminated by the addition of 2 mM Pefabloc, and the homogenate was immunoprecipitated. For the analysis of mAb immunoreactivity against denatured PrP^Sc^, brain homogenate samples were treated according to a previously described method with minor modification [31], [32]. Briefly, brain homogenate samples were mixed with an equal volume of 2% (w/v) SDS and boiled for 10 min. Samples were then diluted in PBS for a final SDS concentration of 0.04% and subjected to the immunoprecipitation described above.

### Western Blotting

The samples were separated by 12% SDS-PAGE and blotted electrically onto a PVDF membrane (Millipore). The blotted membrane was incubated with HRP-conjugated mAb T2 at RT for 1 h. Signals were developed with a chemiluminescent substrate (SuperSignal; Pierce Biotechnology).

### PepSpots Analysis

Epitope analysis was performed according to a previously described method [33]. Briefly, mAbs were incubated with a gridded array of peptides comprising 122 polypeptides of 13 amino acids, shifted by 2 amino acids and covering the entire mouse PrP sequence. The peptides were covalently attached at their COOH-termini to a cellulose membrane as individual spots (JPT Peptide Technologies). Peptide-antibody complexes were detected using HRP-conjugated anti-mouse IgG and chemiluminescent substrate.

### Peptide Competition Assay

Peptide sequences were obtained from mouse PrP sequence. Two peptides, P31–39 (WNTGGSRYP, amino acid sequence positions 31–39 of PrP) and P41–47 (QGSPGGN, positions 41–47 of PrP), were synthesized for peptide competition assays. To examine the specific reactivity of the antibodies to PrP^Sc^, mAbs were incubated with or without 200 µg/mL of synthetic peptide for 16 h at 4°C. Then, antibody-peptide combinations were mixed with the brain homogenate sample [0.05% (w/v)] from BSE-affected mice and immunoprecipitated as described above. Immunoprecipitated sample was subjected to western blotting, and signals were developed with HRP-conjugated mAb T2 and chemiluminescent substrate.

### Histoblot Analysis

Histoblot analysis was performed as described previously [33]. Mouse brains were frozen in liquid N_2_ and then cut into 5 µm sections. The sections were placed on glass slides and immediately pressed onto PVDF membranes. The membranes were thoroughly air-dried and stored at −80°C until use. The blotted membranes were incubated with mAbs. The mAb P2b was used as a negative control, and mAb 31C6 was used as a positive control. MAb binding was detected using HRP-conjugated anti-mouse IgG and chemiluminescence, as described for western blotting.

### Immunohistochemical Analysis

Cryosections with a thickness of 5 µm were fixed with acetone for 10 min and incubated with mAbs without antigen retrieval pretreatment. Immunostaining was performed using a Histofine MAX-PO (M) Kit (Nichirei) with 3, 3′-diaminobenzidine tetrachloride as the chromogen. The sections were then counterstained with hematoxylin.

### Time Course Analysis

The brains of BSE-affected mice were homogenized in 9 volumes of PBS using a multi-bead shocker (Yasui Kikai) and centrifuged at 1,000×*g* for 5 min at RT. The supernatant was then intracerebrally inoculated into female 3-week-old wild-type mice. Mice were sacrificed at 0, 40, 80, and 120 days post-inoculation (dpi) and at the terminal stage (150 days). The brain homogenate [0.05% (w/v)] of each mouse was subjected to western blotting and immunoprecipitation as described above.

## Results

### Generation of PrP^Sc^-specific Antibodies

To generate PrP^Sc^-specific mAbs, we immunized PrP^0/0^ mice with partially purified intact PrP^Sc^. In a primary screening, 33 of 753 wells were positive for native PrP^Sc^. Twelve of thirty-three wells showed stronger reactivity to native PrP^Sc^ than to denatured PrP^Sc^. After further screening and cloning of these 12 wells, we established 2 clones (6A12 and 8D5) that demonstrated stronger reactivity to native PrP^Sc^ than to denatured PrP^Sc^ as candidate PrP^Sc^-specific mAbs (Table 1). Isotype analysis indicated that these mAbs were IgG2b. In contrast, mAb 6H4 exhibited stronger reactivity against denatured PrP^Sc^ than native PrP^Sc^.

**Table 1:** ELISA immunoreactivity of mAbs to native and denatured PrP^Sc.^

mAbs	Isotype	Optical density (O.D. _450 nm_)^a^	
		Gdn (−)	Gdn (+)
6A12	IgG2b	1.15±0.03	0.66±0.06*
8D5	IgG2b	1.22±0.03	0.64±0.05*
6H4	IgG1	0.3±0.02	1.3±0.03*

Gdn (+) denotes the 4M GdnSCN-treated wells (denatured PrP^Sc^). Gdn (−) wells were treated with sterilized water instead of 4M GdnSCN (native PrP^Sc^).

MAbs 6A12 and 8D5 were generated in this study. MAb 6H4 (Prionics) is an anti-PrP antibody that recognizes PrP amino acid residues 143–154.

a Data represent mean optical density (O.D.) ± standard deviation in triplicate experiments.

* Asterisks indicate a statistically significant difference in mAb immunoreactivity between denatured PrP^Sc^ and native PrP^Sc^ (*P*<0.001).

### Specific Immunoreactivity of mAbs 6A12 and 8D5 to PrP^Sc^

**Figure 1:**
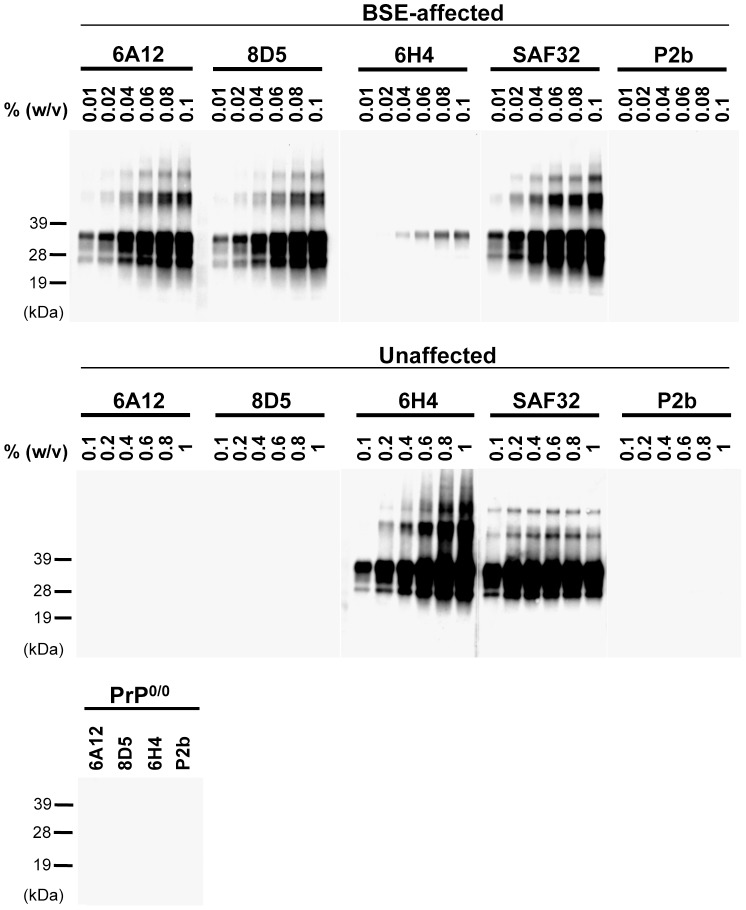
Immunoreactivity of mAbs 6A12 and 8D5 in immunoprecipitation assays. Brain homogenate was incubated with mAb and immunoprecipitated with protein G-coupled magnetic beads. Immunoprecipitated sample was subjected to western blotting and detected by using HRP-conjugated mAb T2. MAbs 6H4 and SAF32 were used as positive controls. MAb P2b, which is isotype-matched with mAbs 6A12 and 8D5, was used as a negative control. The brain homogenates (250 µL) of BSE-affected [0.1–0.01% (w/v)], unaffected [1–0.1% (w/v)], and PrP^0/0^ [1% (w/v)] mice were used. Molecular weight markers are shown on the left (kDa). The concentration of all mAbs for the immunoprecipitation assay was 1 µg/mL. MAbs 6A12 and 8D5 selectivity reacted to PrP from prion-affected individuals.

We performed immunoprecipitation assays to confirm the immunoreactivity of the newly generated mAbs to native PrP^Sc^. The mAbs 6A12 and 8D5 precipitated PrP from the brains of BSE-affected mice [0.1–0.01% (w/v)], but not from those of unaffected mice [1–0.1% (w/v)] (Fig. 1). In contrast, mAb 6H4 strongly recognized PrP from unaffected mice. One mAb, SAF32, which recognizes the octapeptide repeat region as an epitope, reacted equally to PrP in normal and BSE-affected mice (Fig. 1). These results suggest that mAbs 6A12 and 8D5 selectively immunoprecipitated PrP^Sc^ from BSE-affected mice.

**Figure 2:**
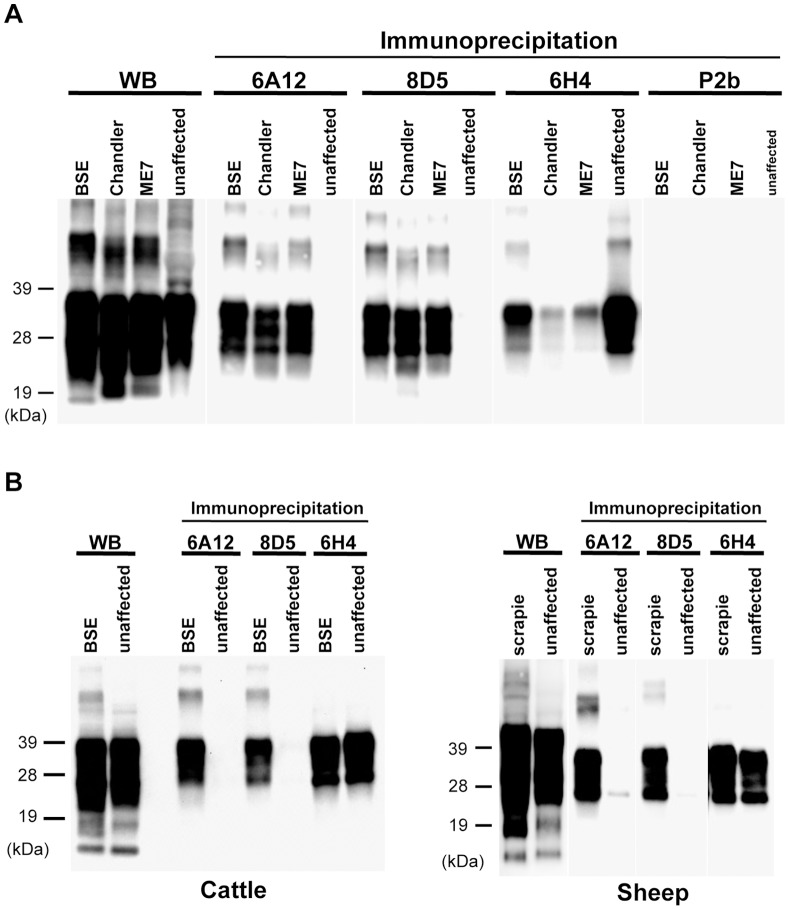
Immunoreactivity of mAbs 6A12 and 8D5 with PrP^Sc^ from different prion strains and animal species. (A) The brain homogenates [0.05% (w/v)] from ME7- and Chandler-affected mice were used. BSE: brain homogenate from BSE-affected mice. (B) Cross-reactivity of mAbs 6A12 and 8D5 with PrP^Sc^ from BSE and scrapie. Brain homogenates from unaffected [1% (w/v)] and affected [0.5% (w/v)] individuals were used. Total PrP in the brain homogenate was detected by routine western blotting (WB). BSE: brain homogenate from C-BSE-affected cattle; Scrapie: brain homogenate from scrapie-affected sheep. Molecular weight markers are shown on the left (kDa). The concentration of all mAbs for the immunoprecipitation assay was 1 µg/mL. MAbs 6A12 and 8D5 cross-reacted with PrP^Sc^ from different prion strains and animal species.

The cross-reactivity of the newly generated mAbs to PrP^Sc^ from other animal species was analyzed. The mAbs 6A12 and 8D5 also detected PrP^Sc^ in the brains of ME7- and Chandler-affected mice (Fig. 2A), as well as PrP^Sc^ in the brains of scrapie-affected hamsters (data not shown), C-BSE-affected cattle, and scrapie-affected sheep (Fig. 2B). No PrP signal from healthy animals was detected (Figs. 2A, B).

### Immunoreactivity of mAbs 6A12 and 8D5 in Histopathology

**Figure 3:**
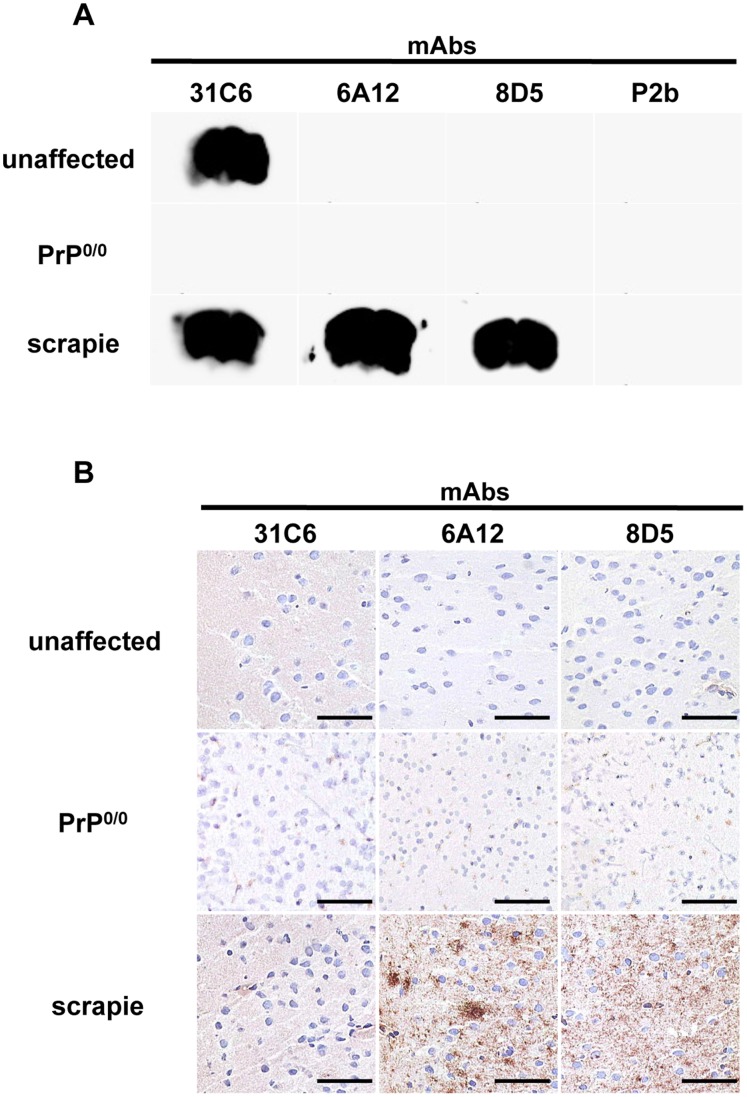
Immunoreactivity of mAbs 6A12 and 8D5 in histopathology. (A) Histoblot analysis. Cryosections of scrapie ME7-affected (scrapie), unaffected, and PrP^0/0^ mouse brains were blotted onto a PVDF membrane. The histoblot membranes were used without autoclaving or denaturing pretreatments. The PrP signal was detected by mAbs 6A12 and 8D5 in scrapie-affected but not unaffected mouse brain. MAb 31C6 reacted with PrP in scrapie-affected and unaffected mouse brain. MAb P2b showed no signal. (B) Immunohistochemical analysis. Cryosections from ME7-affected (scrapie), unaffected, and PrP^0/0^ mouse brains were fixed with acetone. Sections were incubated with primary antibodies without antigen retrieval. Immunostaining was performed using mAbs 31C6, 6A12, and 8D5. The bar represents 50 µm. MAb 31C6 demonstrated a weak PrP signal in unaffected and scrapie-affected mouse brain. In contrast, mAbs 6A12 and 8D5 demonstrated strong PrP signals in scrapie-affected mouse brain but not in unaffected brain.

In histoblot analysis, pan-PrP mAb 31C6 reacted with PrP from unaffected and scrapie-affected mouse brains. MAbs 6A12 and 8D5 demonstrated positive immunoreactivity in brains from scrapie-affected mice brain but not from unaffected mice (Fig. 3A). In acetone-fixed tissue sections, mAb 31C6 reacted faintly with PrP from unaffected and scrapie-affected mice without antigen retrieval. MAbs 6A12 and 8D5 strongly reacted with PrP in scrapie-affected mice, but no signal was detected in unaffected mice (Fig. 3B). These results indicate that mAbs 6A12 and 8D5 specifically react with PrP^Sc^ *in situ*.

### Epitope Analysis of mAbs 6A12 and 8D5

**Figure 4:**
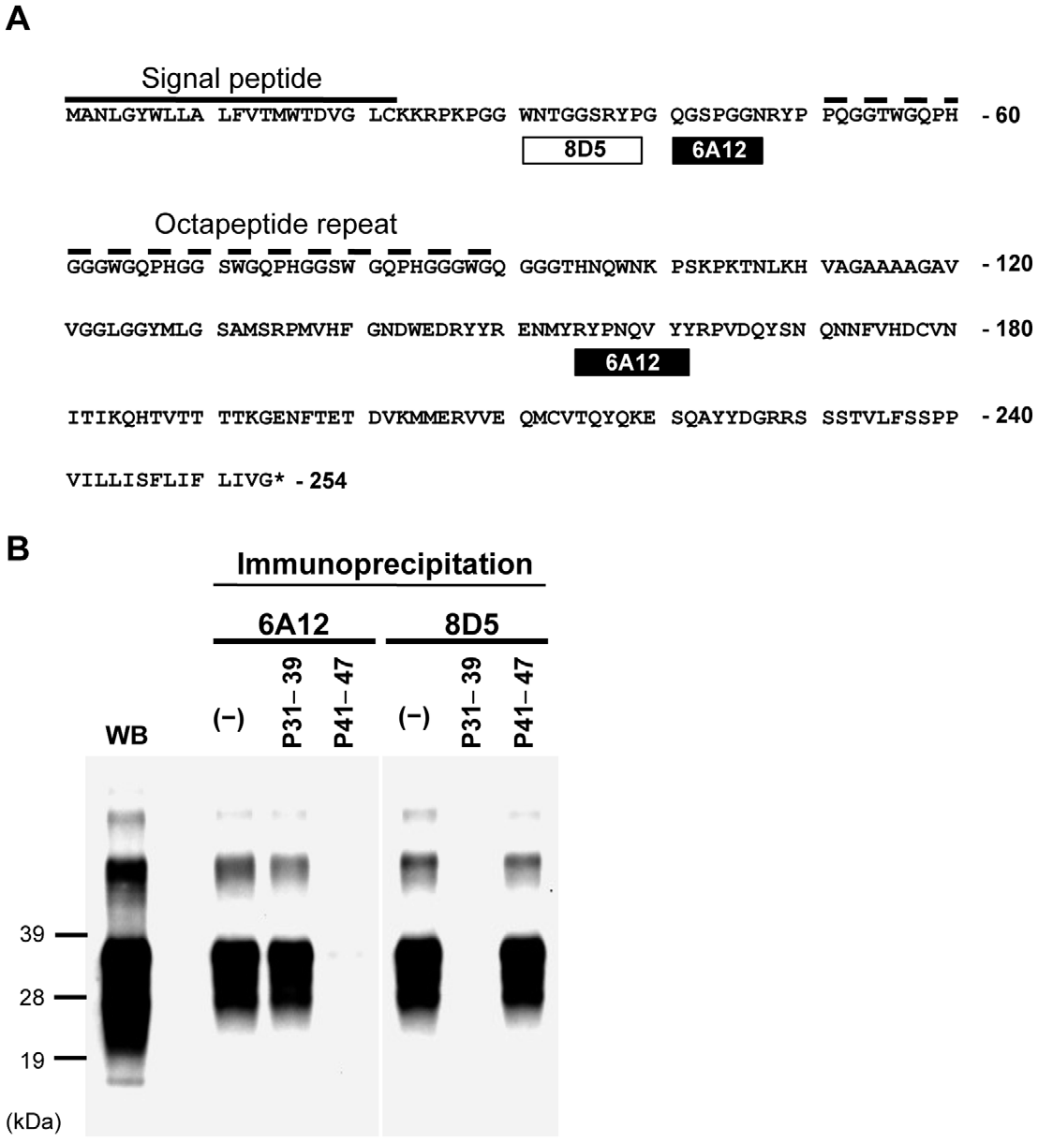
Epitope analysis. (A) The epitopes of 6A12 and 8D5 in the PrP amino acid sequence. The epitope positions of mAbs 6A12 and 8D5 are represented by boxes under the amino acid sequence of mouse PrP (residues 1–254) (Supplemental Fig. 2). The dashed line indicates the octapeptide repeat region. Sequence numbers are shown on the right. (B) Peptide competition assay. P31–39 and P41–47 are synthetic peptides. MAbs were pre-incubated with (P31–39 or P41–47) and without (−) synthetic peptide prior to use in immunoprecipitation. The brain homogenate [0.05% (w/v)] from BSE-affected mice was incubated with the antibody-peptide complex and immunoprecipitated with protein G-coupled magnetic beads. The immunoprecipitated PrP was western blotted and detected with HPR-conjugated mAb T2. Total PrP in the brain homogenate was detected by routine western blotting (WB). Molecular weight markers are shown on the left (kDa). P31–39 blocked the reactivity of mAb 8D5 to PrP^Sc^, but not the reactivity of mAb 6A12 to PrP^Sc^. Conversely, P41–47 blocked the reactivity of mAb 6A12 to PrP^Sc^, while P31–39 did not.

MAbs 6A12 and 8D5 recognized PrP^Sc^ both in solution and *in situ* (acetone-fixed tissues). To elucidate the molecular basis for these observations, we determined the epitopes of these mAbs by a synthetic peptide assay (Fig. S1). MAb 8D5 recognized WNTGGSRYP, at amino acid positions 31–39. MAb 6A12 recognized QGSPGGN, at positions 41–47, and RYPNQVYY, at positions 155–162 (Figs. 4A and S1). To confirm the specific reactivity of the mAbs to PrP^Sc^, we next performed peptide competition assays. We synthesized peptides corresponding to PrP amino acids 31–39 (P31–39) and 41–47 (P41–47). In the peptide competition assays, the homologous synthetic peptide completely blocked the reactivity of mAbs 8D5 and 6A12 to PrP^Sc^, while the heterogeneous peptide did not (Fig. 4B). Further, mAbs 6A12 and 8D5 did not react with PK-digested brain homogenates, which contain PrPcore (Fig. S2). MAb 6A12 also demonstrated faint immunoreactivity against PrP amino acids 155–162 (Figs. 4A and S1) in peptide spots. However, mAb 6A12 did not react with PrPcore (Fig. S2), and the synthetic peptide (P41–47) eliminated the reactivity of mAb 6A12 to PrP^Sc^ (Fig. 4B). This suggests that PrP 155–162 is not essential to the immunoreactivity of mAb 6A12. We next immunoprecipitated denatured PrP^Sc^ to examine the properties of the epitopes for mAbs 6A12 and 8D5 on PrP^Sc^. The brain homogenates of BSE-affected mice were pretreated with SDS (denatured PrP^Sc^) prior to immunoprecipitation. The immunoreactivity of mAb 6H4 against PrP increased after SDS denaturation of the brain samples. MAb SAF32 reacted with PrP in both SDS-denatured and native brain homogenates. In contrast, the immunoreactivity of mAbs 6A12 and 8D5 against PrP^Sc^ was markedly decreased by denaturation (Fig. S3).

### Characterization of PrP^Sc^ Precipitated by mAbs 6A12 and 8D5

**Figure 5:**
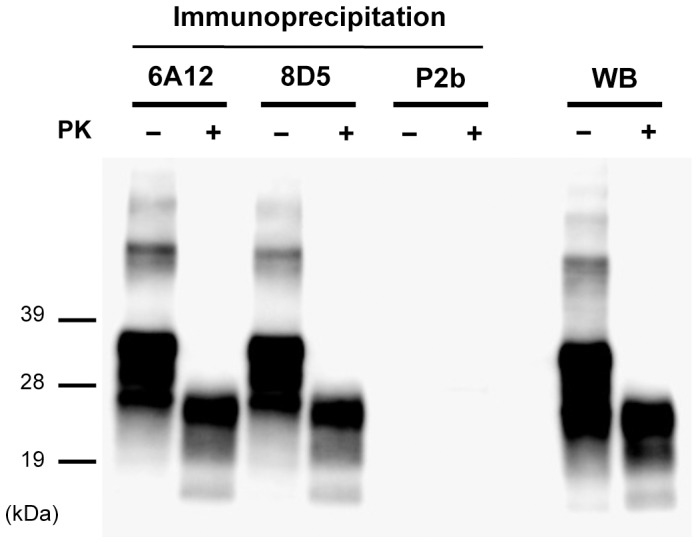
Biochemical analysis of PrP^Sc^ precipitated with the newly generated mAbs. PrP^Sc^ was precipitated by mAbs 6A12 and 8D5 from brain homogenate [0.05% (w/v)] from BSE-affected mice. The precipitated PrP^Sc^ was treated with (+) or without (−) 50 µg/mL of PK for 1 h and then western blotted with mAb T2. PrP signals were detected by HRP-conjugated mAb T2. MAb P2b, which is isotype-matched with mAbs 6A12 and 8D5, was used as negative control. The total amount of PrP and PrPcore in the brain homogenates used in this experiment was detected by routine western blotting (WB). PK treatment decreased the signal intensity of PrP, however, the typical three bands were detected.

To clarify the characteristics of the mAbs, immunoprecipitated PrP^Sc^ was treated with PK. To detect the PrP band in WB, we used mAb T2, which recognizes the PrPcore region. Without PK digestion, precipitated PrP^Sc^ from BSE-affected mice exhibited an intense signal and a molecular weight consistent with that of intact PrP. In contrast, with PK treatment, precipitated PrP^Sc^ from BSE-affected mice showed a shifted molecular weight consistent with that of the PrPcore, and its signal intensity was decreased (Fig. 5). These results suggest that mAbs 6A12 and 8D5 isolate intact PrP^Sc^, including the PK-resistant isoform of PrP^Sc^, from prion-affected individuals.

### Native PrP^Sc^ Dynamics in Prion Disease

**Figure 6:**
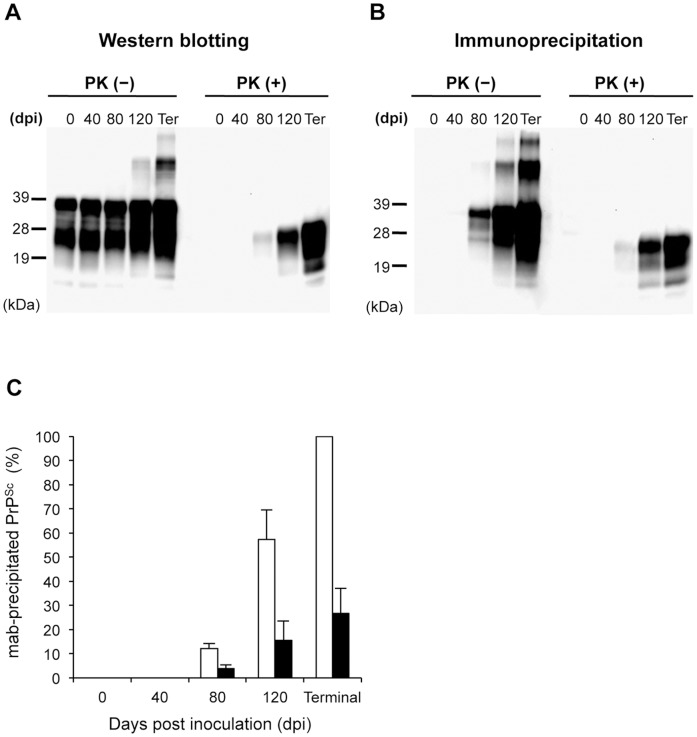
Dynamics of PrP^Sc^ in BSE-affected mice. BSE-affected mice were killed at 0, 40, 80, 120, and 150 dpi (terminal stage). (A) PrP detection by routine western blotting in disease stages. Brain homogenate [0.05% (w/v)] from BSE-affected mice was treated with (+) or without (−) 50 µg/mL PK for 1 h followed by western blotting with mAb T2. (B) PrP^Sc^ detection by immunoprecipitation. PrP^Sc^ was precipitated by mAb 6A12 from the same samples as in (A). The precipitated PrP^Sc^ was treated with (+) or without (−) PK, and PrP signals were detected by HRP-conjugated mAb T2. PK (−) samples indicate the total amount of PrP^Sc^, and PK (+) samples represent the amount of PrPcore. Molecular weights are shown on the left (kDa). (C) Quantification of PrP^Sc^ signals in (B). The band intensity relative to total PrP^Sc^ (%) at the terminal stage is shown. Black bar: PrPcore. White bar: total PrP^Sc^. All values were calculated as the mean ± standard deviation of at least 3 independent experiments.

We successfully isolated native and intact PrP^Sc^ by using the newly generated mAbs. Next, we examined the dynamics of native PrP^Sc^ in BSE-affected mice over the disease time course using these mAbs. PrP^Sc^ signals were detected at 80 dpi by routine western blotting (Fig. 6A). In immunoprecipitation assays with mAb 6A12, the PrP^Sc^ signal was detected at 80, 120, and 150 dpi (Fig. 6B). The dynamics of PrPcore detected after PK digestion of 6A12-precipitated PrP^Sc^ were similar to those of PrPcore detected by western blotting (Figs. 6A, B). To estimate the amount of PrPcore relative to the amount of total PrP^Sc^, the signal intensity of 6A12-precipitated PrP^Sc^ with PK digestion (PrPcore) was compared to that of 6A12-precipitated PrP^Sc^ without PK digestion (total PrP^Sc^). The ratios of PrPcore to total PrP^Sc^ were 3.9∶12.2, 15.4∶57.7, and 27.0∶100 at 80, 120, and 150 dpi, respectively (Fig. 6C). Interestingly, the percentage of PrPcore was constant (27–30% of total PrP^Sc^) throughout the course of the disease.

## Discussion

To gain insight into native PrP^Sc^, we attempted to generate PrP^Sc^-specific antibodies by immunizing PrP-deficient mice with intact PrP^Sc^ from BSE-affected mice. With this approach, we obtained novel PrP^Sc^-specific mAbs, named 6A12 and 8D5, which recognized the PrP amino acids 31–39 and 41–47 as their epitopes, respectively. Interestingly, neither mAb recognized PrP^C^ or SDS-denatured PrP^Sc^ in solution. These results indicate that this region may be refolded or that another molecule may bind to PrP^C^ in solution, thus burying the epitopes. In contrast, the primary sequence was exposed in PrP^Sc^ in solution and *in situ*. Furthermore, mAbs 8D5 and 6A12 cross-reacted with PrP^Sc^ of different animal species, indicating that a PrP^Sc^-specific epitope exists in the N-terminal region of PrP^Sc^ that is conserved across prion strains and animal species.

Previously reported PrP^Sc^-specific mAbs recognize epitopes putatively located in the PrPcore or C-terminal region of PrP^Sc^ [15], [17], [18], [20]. MAb 15B3 reacted specifically with PrP^Sc^ by recognizing three different peptide segments (at 142–148, 162–170, and 214–226) between 2 PrP molecules [15]. MAb 6H10 recognized a conformational epitope on PrP^Sc^ in the C-terminal region [18]. Furthermore, the PrP^C^-PrP^Sc^ binding motif was identified on PrP^Sc^ using genetically-produced PrP (mouse sequence residues 89–112 and 136–158) motif-grafted antibodies [34], [35]. Additionally, a YYR motif in PrP (human sequence residues 149–151, 162–164, and 225–227) was reported to be a conformation-selective linear epitope of PrP^Sc^ [16]. In this study, we also identified a novel conformation-selective linear epitope in the N-terminal region of PrP^Sc^ (mouse PrP 31–39 and PrP 41–47).

The N-terminal domain of PrP consists of positively charged amino acid sequences and an octapeptide repeat sequence [5]. The octarepeat domain may bind copper ions [36] and affect copper metabolism [37]. Extra octarepeats resulted in inherited prion diseases in humans [38], and recombinant PrP containing additional octapeptide repeats displays detergent insolubility and resistance to PK digestion similar to PrP^Sc^ [39]. It has reported that the N-terminus structure of PrP was changed with pathogenic mutations, and it has linked to the binding ability with glycosaminoglycan, which was thought to be involved in prion pathogenesis [40]–[42]. Furthermore, a recent report showed the presence of a PrP^Sc^-specific conformation in the octarepeat domain using trypsin digestion methods [43]. These results suggest that the N-terminal region of PrP^Sc^ is important for prion pathogenesis. Recently, recombinant PrP phosphorylated at serine 43 was reported to exhibit a conformation change that conferred proteinase resistance and aggregate formation [44]. The epitope of PrP^Sc^-specific mAb 6A12 includes this residue, and this mAb will be a powerful tool with which to clarify the mechanism of this conformational change.

PrP^Sc^-sen has been demonstrated by a conformation dependent immune assay [12], [45], [46], cold PK digestion [47], immunoblotting following biochemical purification [13], [14], immunologic detection [48], and thermolysin digestion [46], [49]. However, no information on the dynamics PrP^Sc^-sen throughout the disease time course has been previously reported. The time course experiment in the present study addressed this question. PrP^Sc^-sen is a protease-sensitive isoform of native PrP^Sc^ [12]. Indeed, we observed a decrease in PrP^Sc^ signal intensity after PK digestion, and this decreased signal intensity was thought to result from the disappearance of PrP^Sc^-sen. We estimated the relative amount of PrP^Sc^-sen by comparing the PrP^Sc^ signal intensities before and after PK digestion. PrPcore was detected from 80 days post-inoculation by western blotting, and PrP^Sc^-sen was detected simultaneously. The relative amount of PrP^Sc^-sen was constant (70–73% of total PrP^Sc^) throughout the disease time course. This may indicate an association of PrP^Sc^-sen with PrP^Sc^ generation and/or degradation. Further study is needed to clarify the metabolism of PrP^Sc^.

PrP^Sc^-sen was estimated to comprise approximately 90% of total PrP^Sc^ in prion-affected individuals at the terminal stage by thermolysin experiments [49] and conformation-dependent immune assays [45], [46]. The present study estimated the proportion of PrP^Sc^-sen to be approximately 70%. In this study, we isolated PrP^Sc^-sen on the basis of a PrP^Sc^-specific epitope at the N-terminal region. The structural selection of PrP^Sc^ by the mAb may account for the difference.

The BSE prion has different characteristics than scrapie. The molecular weight of PrPcore from BSE is smaller than that from scrapie, and this difference is thought to derive from their different structures and/or conformations [22]. In previous reports, scrapie mouse-PrP^Sc^–specific mAbs were generated by immunizing intact PrP^Sc^ from scrapie mice [20]. In this study, we could not acquire BSE-PrP^Sc^–specific mAbs from mice immunized with intact PrP^Sc^. However, we identified a novel PrP^Sc^-specific epitope located at the N-terminal region of PrP. These findings suggest that multiple PrP^Sc^-specific epitopes exist on PrP^Sc^; this approach could help establish a panel of PrP^Sc^-specific antibodies. PrP^Sc^-specific mAbs 6A12 and 8D5 are expected to be powerful tools for the analysis of native and intact PrP^Sc^, which will lead to advances in understanding the pathogenesis of prion diseases.

## Supporting Information

Figure S1 **Epitope mapping by peptide array.** MAbs were reacted with a peptide array on a cellulose membrane. The gridded array of peptides comprised 122 polypeptides of 13 amino acids that shifted by 2 amino acids and covered the entire mouse PrP sequence. The reactivity of the mAbs to the peptide spots was detected using HRP-conjugated anti-mouse IgG and chemiluminescent substrate. MAb 6A12 reacted with synthetic peptides No. 18–21 and No. 76–78. MAb 8D5 reacted with No. 14–16. MAb P2b, which recognizes a plant-derived protein, was used as a negative control. No signal was detected in peptide assay using MAb P2b.
(TIF)

Figure S2 **Immunoreactivity of mAbs 6A12 and 8D5 against PK-digested PrP^Sc^ (PrPcore) in immunoprecipitation assays.** Brain homogenate was treated with (+) or without (−) PK and immunoprecipitated. MAb P2b was used as a negative control. Immunoprecipitated PrP were detected by western blotting with HRP-conjugated mAb T2. The total amount of PrP in the brain homogenates was detected by routine western blotting. B: brain homogenates [0.05% (w/v)] from BSE-affected mice; N: brain homogenates [0.3% (w/v)] from unaffected mice. Neither of the generated mAbs reacted to the PK-digested PrP^Sc^.
(TIF)

Figure S3 **Immunoreactivity of mAbs 6A12 and 8D5 to denatured PrP^Sc^.** Brain homogenates from BSE-affected mice were mixed with an equal volume of 2% (w/v) SDS and boiled. Sample were then diluted in PBS for a final SDS concentration of 0.04% (refolding), immunoprecipitated, and western blotted. The total PrP in each homogenate was detected by routine western blotting (WB). SDS (+): SDS-denatured brain sample; SDS (−): native brain sample. The immunoreactivity of mAb 6H4 against PrP was increased with SDS denaturation. MAb SAF32 reacted with PrP in both SDS-denatured and native brain homogenates. In contrast, the immunoreactivity of mAbs 6A12 and 8D5 against PrP^Sc^ was markedly decreased by SDS denaturation.
(TIF)
