# Supplementary figures and images for: The N-Terminal Sequence of Prion Protein Consists an Epitope Specific to the Abnormal Isoform of Prion Protein (PrPSc)
Source: PLoS One. 2013 Feb 28;8(2):e58013. doi: 10.1371/journal.pone.0058013 (PMC3585212; doi:10.1371/journal.pone.0058013)

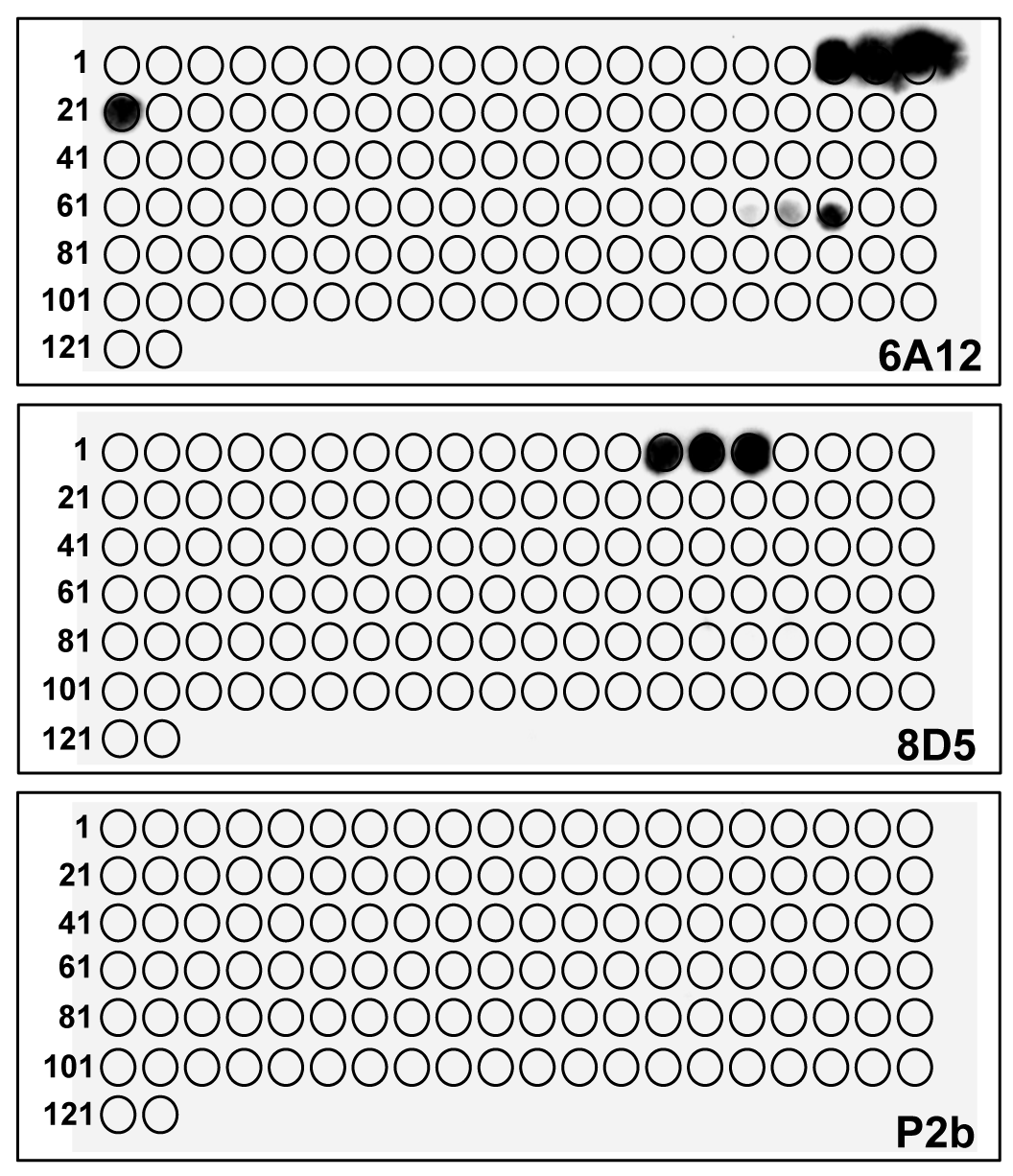

Supplement: Figure S1 — Epitope mapping by peptide array. MAbs were reacted with a peptide array on a cellulose membrane. The gridded array of peptides comprised 122 polypeptides of 13 amino acids that shifted by 2 amino acids and covered the entire mouse PrP sequence. The reactivity of the mAbs to the peptide spots was detected using HRP-conjugated anti-mouse IgG and chemiluminescent substrate. MAb 6A12 reacted with synthetic peptides No. 18–21 and No. 76–78. MAb 8D5 reacted with No. 14–16. MAb P2b, which recognizes a plant-derived protein, was used as a negative control. No signal was detected in peptide assay using MAb P2b. (TIF) [file pone.0058013.s001.tif]

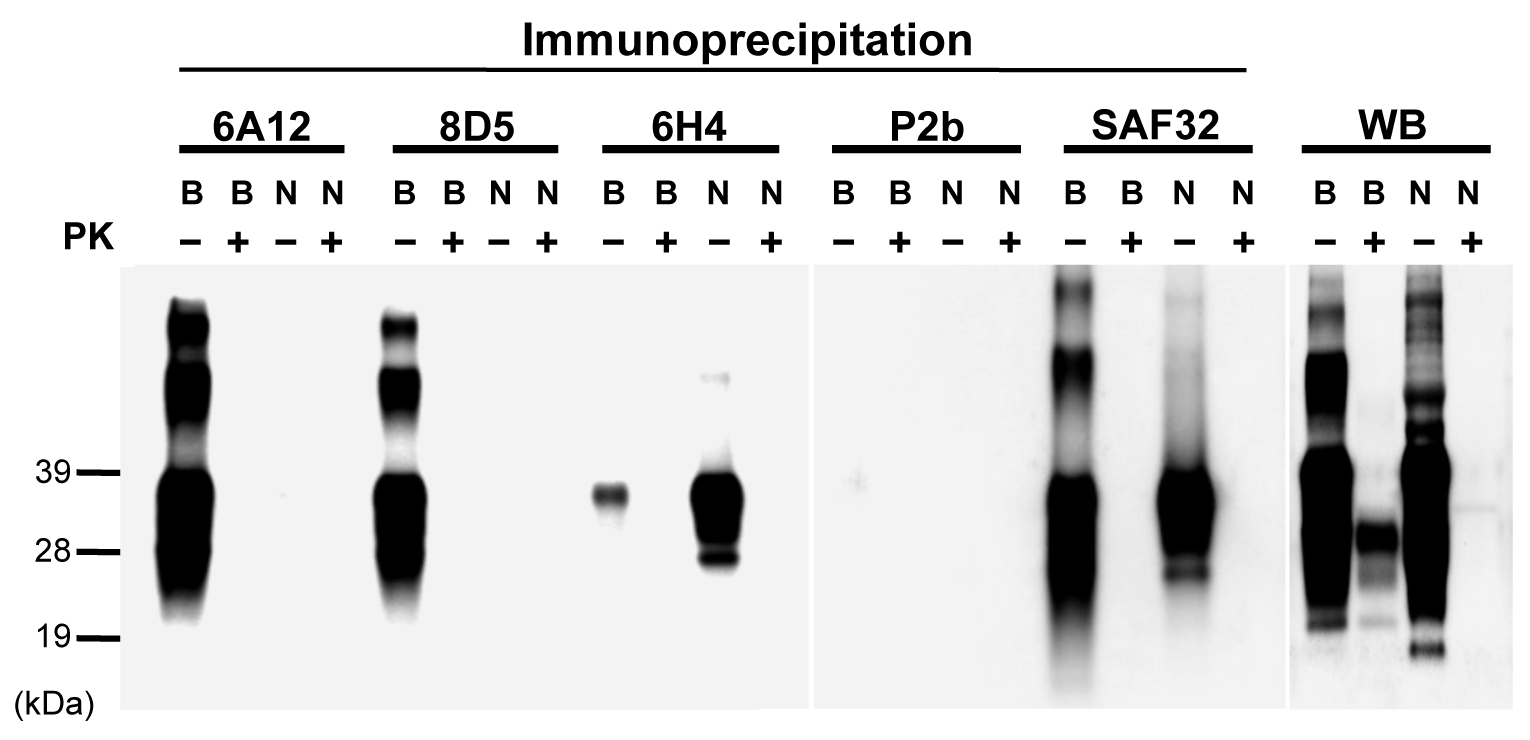

Supplement: Figure S2 — Immunoreactivity of mAbs 6A12 and 8D5 against PK-digested PrPSc (PrPcore) in immunoprecipitation assays. Brain homogenate was treated with (+) or without (−) PK and immunoprecipitated. MAb P2b was used as a negative control. Immunoprecipitated PrP were detected by western blotting with HRP-conjugated mAb T2. The total amount of PrP in the brain homogenates was detected by routine western blotting. B: brain homogenates [0.05% (w/v)] from BSE-affected mice; N: brain homogenates [0.3% (w/v)] from unaffected mice. Neither of the generated mAbs reacted to the PK-digested PrPSc. (TIF) [file pone.0058013.s002.tif]

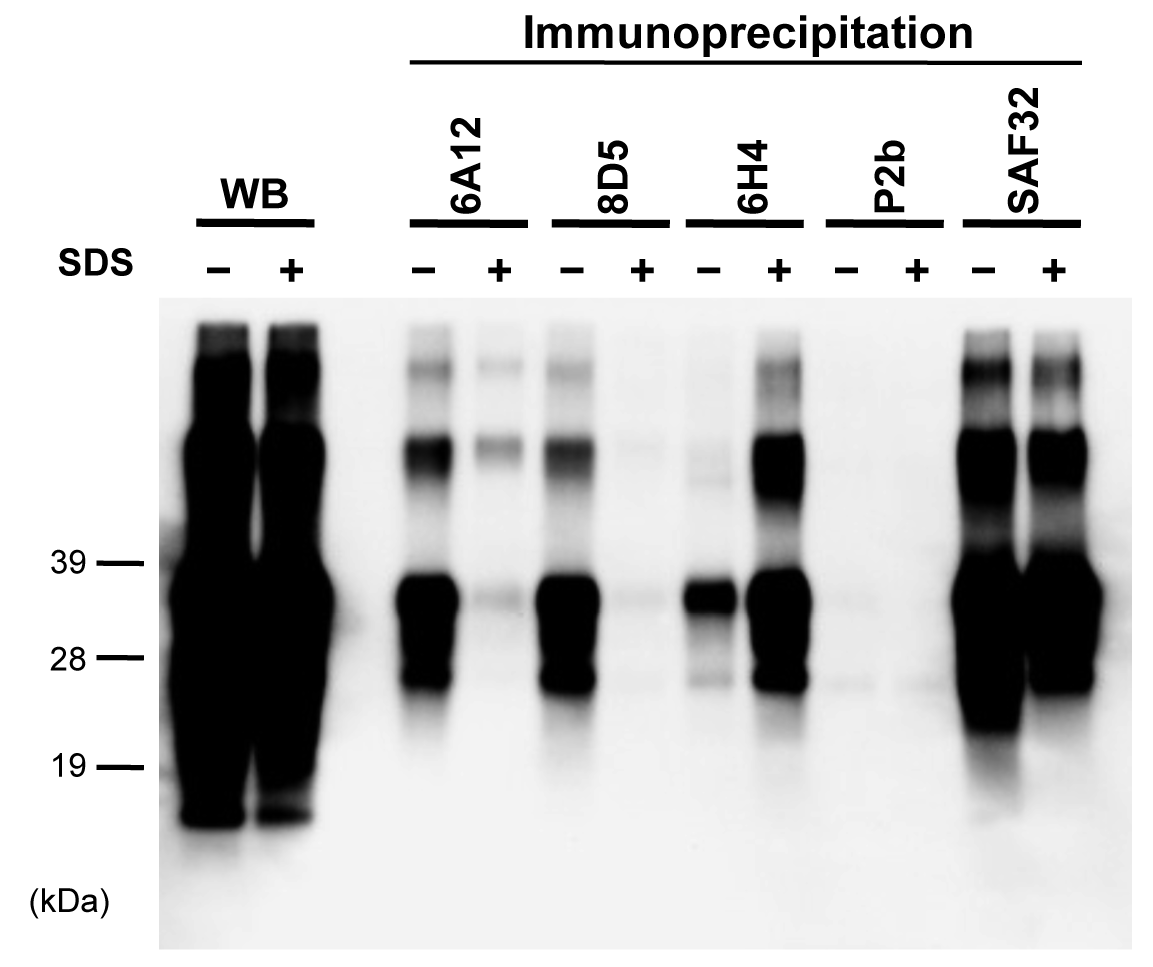

Supplement: Figure S3 — Immunoreactivity of mAbs 6A12 and 8D5 to denatured PrPSc. Brain homogenates from BSE-affected mice were mixed with an equal volume of 2% (w/v) SDS and boiled. Sample were then diluted in PBS for a final SDS concentration of 0.04% (refolding), immunoprecipitated, and western blotted. The total PrP in each homogenate was detected by routine western blotting (WB). SDS (+): SDS-denatured brain sample; SDS (−): native brain sample. The immunoreactivity of mAb 6H4 against PrP was increased with SDS denaturation. MAb SAF32 reacted with PrP in both SDS-denatured and native brain homogenates. In contrast, the immunoreactivity of mAbs 6A12 and 8D5 against PrPSc was markedly decreased by SDS denaturation. (TIF) [file pone.0058013.s003.tif]
